# Supplementary material for: The DNA adenine methylase of Salmonella Enteritidis promotes their intracellular replication by inhibiting arachidonic acid metabolism pathway in macrophages
Source: Front Microbiol. 2023 Mar 2;14:1080851. doi: 10.3389/fmicb.2023.1080851 (PMC10018194; doi:10.3389/fmicb.2023.1080851)
Supplement: Supplementary file 2 [file Data_Sheet_2.zip › S1 Appendix. Non-targeted metabolomics raw data/4.MetDiffAnalysis/C50336_WT.vs.control/C50336_WT.vs.control_neg_corr.pdf]

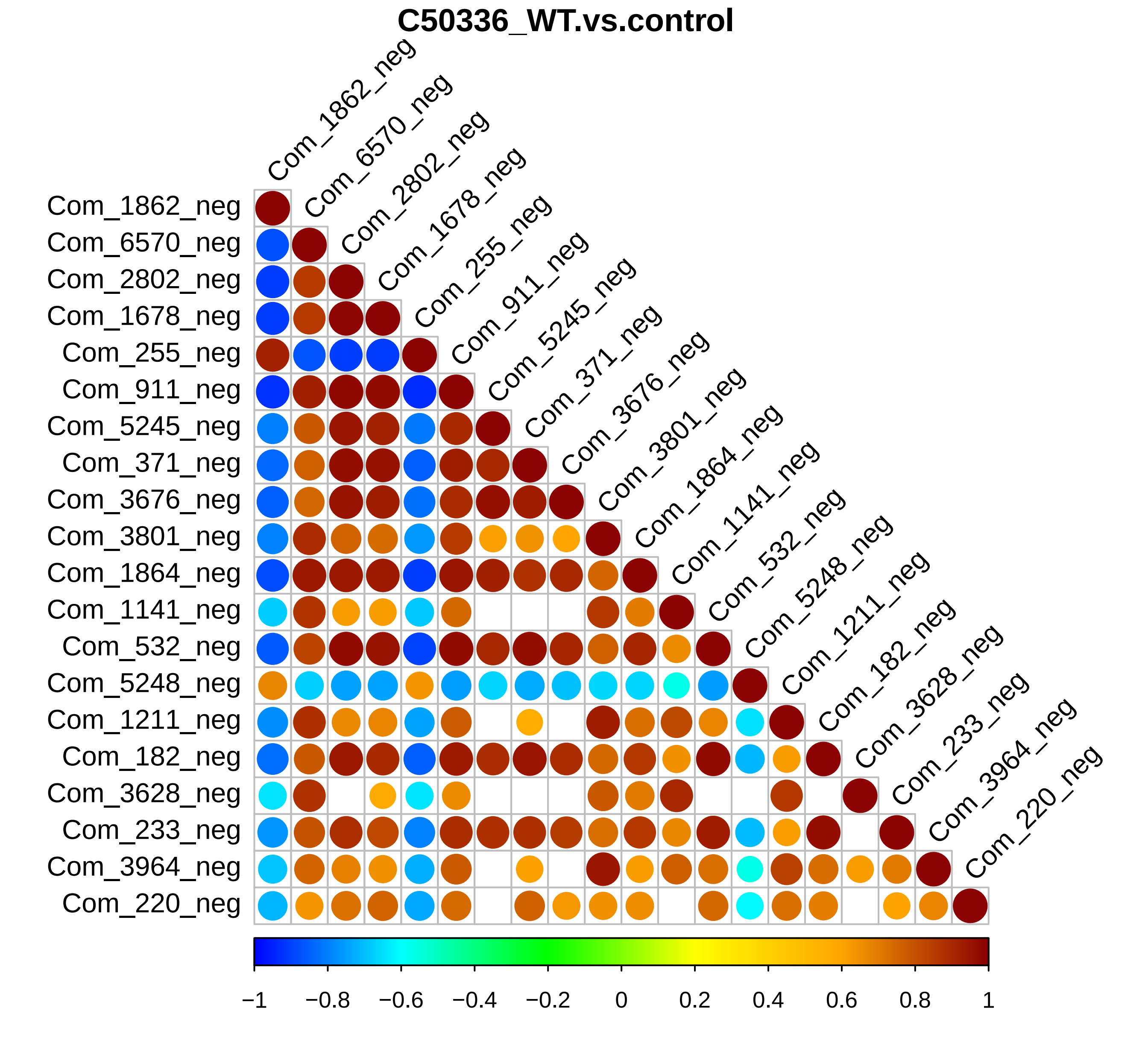
C50336_WT.vs.control
Com_1862_neg
Com_6570_neg
Com_2802_neg
Com_1678_neg
Com_255_neg
Com_911_neg
Com_5245_neg
Com_371_neg
Com_3676_neg
Com_3801_neg
Com_1864_neg
Com_1141_neg
Com_532_neg
Com_5248_neg
Com_1211_neg
Com_182_neg
Com_3628_neg
Com_233_neg
Com_3964_neg
Com_220_neg
−1
−0.8
−0.6
−0.4
−0.2
0
0.2
0.4
0.6
0.8
1
